# Ecology, biology and distribution of spotted-fever tick vectors in Brazil

**DOI:** 10.3389/fcimb.2013.00027

**Published:** 2013-07-12

**Authors:** Matias P. J. Szabó, Adriano Pinter, Marcelo B. Labruna

**Affiliations:** ^1^Laboratório de Ixodologia, Faculdade de Medicina Veterinária, Universidade Federal de UberlândiaUberlândia, Brazil; ^2^Núcleo de Estudos de Doenças Transmitidas por Carrapatos, Superintendência de Controle de EndemiasSão Paulo, Brazil; ^3^Departamento de Medicina Veterinária Preventiva e Saúde Animal, Faculdade de Medicina Veterinária e Zootecnia, Universidade de São PauloSão Paulo, Brazil

**Keywords:** ecology, Brazil, tick species, *Rickettsia*, human spotted-fever

## Abstract

Spotted-fever-caused *Rickettsia rickettsii* infection is in Brazil the major tick-borne zoonotic disease. Recently, a second and milder human rickettsiosis caused by an agent genetically related to *R. parkeri* was discovered in the country (Atlantic rainforest strain). Both diseases clearly have an ecological background linked to a few tick species and their environment. Capybaras (*Hydrochoerus hydrochaeris*) and *Amblyomma cajennense* ticks in urban and rural areas close to water sources are the main and long-known epidemiological feature behind *R. rickettsii*-caused spotted-fever. Unfortunately, this ecological background seems to be increasing in the country and disease spreading may be foreseen. Metropolitan area of São Paulo, the most populous of the country, is embedded in Atlantic rainforest that harbors another important *R. rickettsii* vector, the tick *Amblyomma aureolatum*. Thus, at the city–forest interface, dogs carry infected ticks to human dwellings and human infection occurs. A role for *R. rickettsii* vectoring to humans of a third tick species, *Rhipicephalus sanguineus* in Brazil, has not been proven; however, there is circumstantial evidence for that. A *R. parkeri*-like strain was found in *A. ovale* ticks from Atlantic rainforest and was shown to be responsible for a milder febrile human disease. *Rickettsia*-infected *A. ovale* ticks are known to be spread over large areas along the Atlantic coast of the country, and diagnosis of human infection is increasing with awareness and proper diagnostic tools. In this review, ecological features of the tick species mentioned, and that are important for *Rickettsia* transmission to humans, are updated and discussed. Specific knowledge gaps in the epidemiology of such diseases are highlighted to guide forthcoming research.

## Introduction

Spotted-fever group (SFG) rickettsiae are chiefly transmitted by ticks and may cause mild to severe human infectious disease. These agents are found worldwide and are transmitted by an array of tick species, each one with specific ecological requirements. Thus, epidemiology of the various rickettsioses is determined by specific vector tick geographic and micro environmental distribution.

Brazil is a country with continental dimensions and encompasses diverse biomes such as rainforests (Amazonic and Atlantic), savannah (Cerrado), open fields (Pampas), semi-arid areas (Caatinga), and floodplains (Pantanal). A both numerous and rich fauna is superimposed to these ecological assembly, including 64 tick species (Dantas-Torres et al., 2009, 2012; Labruna and Venzal, 2009; Nava et al., 2010a). Furthermore, it also upholds a diverse microbiota together with *Rickettsia* spp. Curiously, until 2000 only one SFG, *Rickettsia rickettsii*, was known in the country but during the last 12 years this number jumped to five with the inclusion of *R. parkeri, R. rhipicephali, R. amblyommii*, and *R. felis* (Labruna et al., 2011a).

Whereas spotted-fever-caused *R. rickettsii* infection is in Brazil the major and long-known human tick-borne disease (Magalhães, 1952; Lemos et al., 1994; Angerami et al., 2012), only recently was a second and milder tick-borne SFG human rickettsiosis discovered (Spolidorio et al., 2010). Such late discovery is linked to recent use of more appropriate diagnostic tools for *Rickettsia* and an increased research of tick ecology in the country. These more contemporary requirements for diagnosis are understandable if one considers that human rickettsiosis in Brazil is most of the time overshadowed by several other febrile illnesses. Thus, diseases such as dengue fever, common flu, leptospirosis, meningococical meningitis, and others are blamed for or delay diagnosis of human rickettsiosis. In fact, ecological background linked to vector tick species and their environment is of utter importance to provide the first and many times the sole information for timely diagnosis and effective treatment of human patients.

We herein update ecology, biology, and distribution of spotted-fever tick vectors in Brazil and that are important for *Rickettsia* transmission to humans. Specific knowledge gaps in the epidemiology of such diseases are highlighted to guide forthcoming research.

## *Rickettsia rickettsii*—brazilian spotted-fever (BSF)

**Figure 1:**
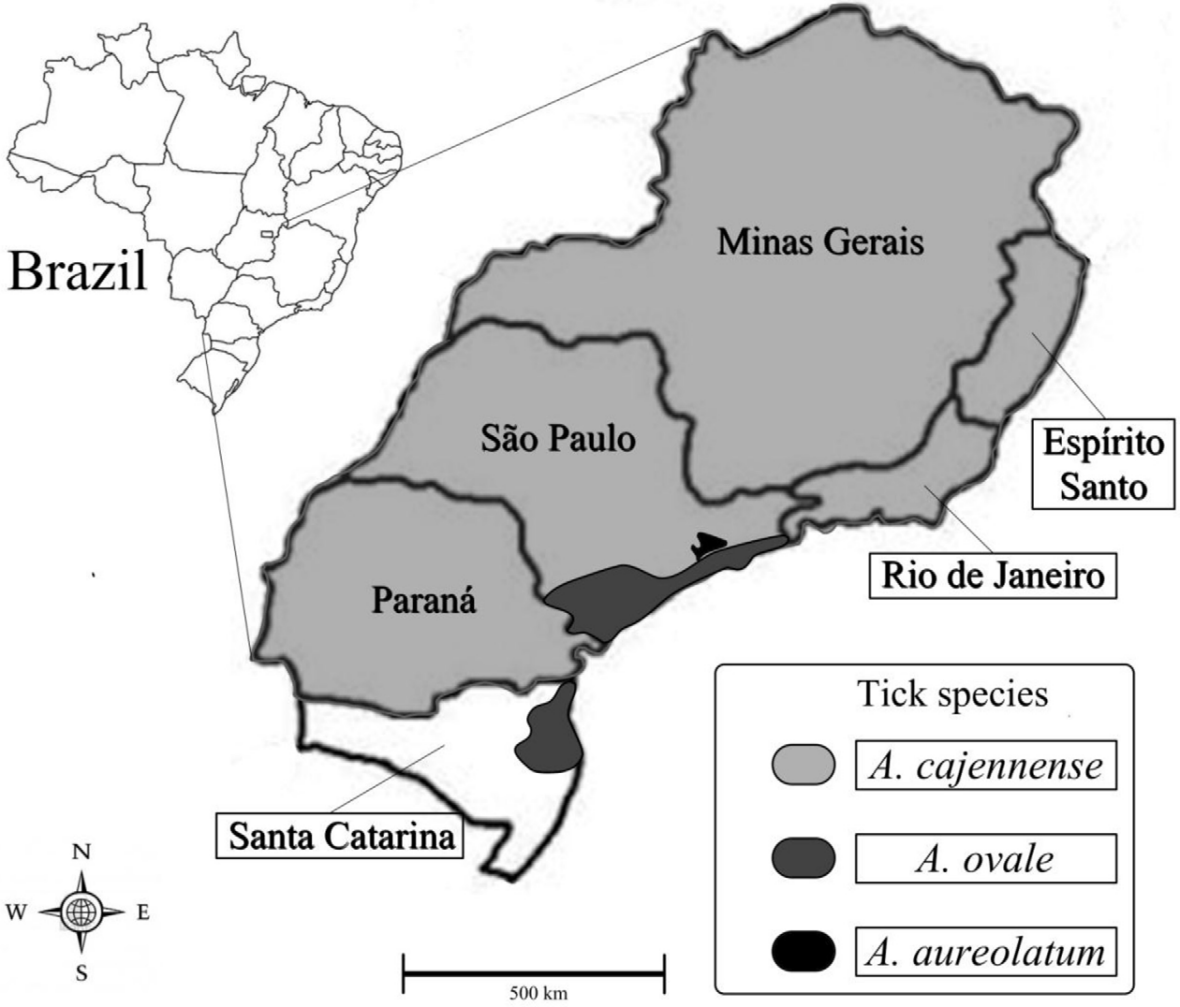
**Locations of spotted-fever vector ticks in Brazil**. Light gray: States from Brazil where *A. cajennense* is proven or suspected vector of *R. rickettsii* to humans. Dark gray: areas where Atlantic rainforest *Rickettsia*-infected *A. ovale* were already found. Black: area with *A. aureolatum* transmission of *R. rickettsii* to humans.

Spotted-fever by *R. rickettsii* infection has been reported in Brazil since the 1920s and is caused by the same agent of the North American Rocky Mountain spotted-fever (reviewed by Labruna, 2009). However, vector tick species in Brazil are different and thus epidemiology of *R. rickettsii*-caused spotted-fever is quite different from that of North America. Hence, the frequent name in publications “BSF.” BSF is a severe, acute disease and fatality rates are between 30 and 40% (Angerami et al., 2006). The disease is more frequently reported in the southeastern region of the country, encompassing the states of Minas Gerais, Rio de Janeiro, São Paulo, Espírito Santo, and Paraná (Figure 1). The highest incidence has occurred in São Paulo, the most populous Brazilian state, where during 2012 there were 68 confirmed cases that resulted in 37 fatalities (54% fatality rate), highlighting BSF absolutely as the vector-borne disease with highest fatality rate in southeastern Brazil (São Paulo Public Health Department).

There has been a clear reemergence of BSF since of the end of 1980s and ecological factors seem to play a major role in that (Labruna, 2009). Undoubtedly, tick vectors are a central link between the *Rickettsia* source and humans and thus tick ecology is basis for BSF epidemiology. In Brazil two tick species, *Amblyomma cajennense,* and *A. aureolatum*, are considered main vectors of BSF whereas *Rhipicephalus sanguineus* is a suspected vector, and may play a role in transmission in particular situations.

## Amblyomma cajennense

In southeast Brazil, location of most BSF cases, the disease occurs chiefly within a well-known ecological background: high *A. cajennense* environmental infestations maintained by one of its major hosts, the capybaras (*Hydrochoerus hydrochaeris*). At the same time, this background is far from being understood and several features are unknown.

*A. cajennense* is a tick species with wide distribution within the Neotropical region with tick populations from Southern Texas, USA, to South of South America as far as latitude 29°S (Estrada-Peña et al., 2004). However, care must be taken with this alleged geographical range. It has been recently proposed that this tick species is in fact a complex of species (Labruna et al., 2011b; Mastropaolo et al., 2011) and BSF vectoring by what is known today as *A. cajennense* probably differs according to tick populations. It is thus important to clearly define the tick species from the *A. cajennense* complex of southeast Brazil. From here on, information provided will refer solely to this specific tick population from southeast Brazil.

*A. cajennense* is a three-host tick species (Guglielmone et al., 2006a) and high environmental infestations in southeast Brazil are associated to hosts such horses and capybaras which feed the more host specific adult stages as well (Labruna et al., 2001; Oliveira et al., 2003; Heijden et al., 2005; Pacheco et al., 2007). This tick species has a 1-year life cycle (Serra Freire, 1982; Labruna et al., 2002; Oliveira et al., 2003) driven by a behavioral diapause of larvae (Labruna et al., 2003). Although egg hatching may occur in summer, larvae seek for hosts only in autumn, a behavior triggered by decrease of day length and temperature (Cabrera and Labruna, 2009). Thus, adults predominate in spring and summer, larvae in autumn and winter, and nymphs in winter and spring. Most BSF cases occur during nymph season (Pinter et al., 2011). Although other factors may be involved, high aggressiveness of nymphs to humans, smaller size that many times precludes host awareness, and a wider spread over the infested area are related to such seasonality of *A. cajennense*-vectored BSF. In addition, laboratory experiments have shown low vector competence of *A. cajennense*-infected larvae, contrasting to high vector competence of infected nymphs (Soares et al., 2012).

Many ecological features of *A. cajennense* from southeast Brazil are unknown, but a few observations indicate that it is being favored by anthropogenic factors. Should this tick species have adequate host supply, it thrives in green areas with at least small amount of shadow provided by herbaceous vegetation or small trees. Labruna et al. (2001), for example, observed at stud farms that presence of *A. cajennense* was statistically associated with the presence of at least one mixed overgrowth pasture (presence of undesired plants such as bushes and shrubs in pasture). This tick species is frequently found associated to riparian forests close to human settlements (Souza et al., 2006) as well. In fact such riparian forests are preferred habitats of capybaras, and a frequent place for contact with humans seeking for leisure.

Under natural conditions, *A. cajennense* is a tick species associated to the Cerrado biome, the Brazilian savannah (Knight, 1992; Szabó et al., 2007a; Veronez et al., 2010). At the same time it is generally absent from the Atlantic rainforest, but it will soon appear at degraded areas of such Biome (Szabó et al., 2009). Within the Cerrado, *A. cajennense* is more linked to forestall phytophisiognomies (Veronez et al., 2010) probably to avoid desiccation. On the other way round, high humidity of rainforests seems to be deleterious to this tick species (Labruna et al., 2005a; Szabó et al., 2009).

Capybara (*H. hydrochaeris*), the largest living rodent, is a semi-aquatic and gregarious species and is widely distributed in South America (review by Pachaly et al., 2001). This rodent is a suitable host for *A. cajennense* ticks and capybara populations are associated to high environmental infestations (Heijden et al., 2005; Souza et al., 2006; Perez et al., 2008; Queirogas et al., 2012). Moreover, this host was experimentally proven to be an as amplifier host of *R. rickettsii* for *A. cajennense* ticks (Souza et al., 2009). Populations of this rodent have increased in southeastern Brazil, notably in human-altered landscape. According to Ferraz et al. (2007), capybaras populations expanded in such areas favored by various factors such as hunting prohibition by Brazilian federal law, high reproductive capacity, decline in natural predators, and rising agricultural production and which provides food. Unfortunate coincidence provided adequate habitat for both capybaras and *A. cajennense* ticks at human-altered landscape. Thus, both are abundant close to human settlements in riparian forests and at habitats with water bodies such as urban and peri-urban parks, garden of condominiums, companies, and similar.

*R. rickettsii* can be found in *A. cajennense* ticks in endemic areas (Guedes et al., 2005), but it is a rare event (Sangioni et al., 2005; Pacheco et al., 2009). Experimental data shows *that A. cajennense* is inadequate host for *R. rickettsii*. This tick species has a low efficiency to maintain the bacterium through successive generations, and *R. rickettsii* infection rates of ticks decline drastically throughout the successive tick generations (Soares et al., 2012). At the same time, Souza et al. (2009) demonstrated that *R. rickettsii* could infect capybaras without causing clinical illness and that rickettsemia for, approximately 10 days, was capable to infect ticks. Thus, capybaras can act as amplifier host of *R. rickettsii* in *A. cajennense* ticks populations in Brazil but a continuous supply of *Rickettsia*-naive capybaras (usually juvenile animals) is needed for a regular creation of new lineages of infected ticks. In this regard, it is intriguing that BSF is endemic in a few locations but absent in others with high populations of both capybara and *A. cajennense*. Therefore, other and unknown factors also play a role to establish or restrain endemicity.

## Amblyomma dubitatum

Capybaras are considered the main host for all stages of another three-hosted tick, *A. dubitatum* Neumann, 1899 (=*A. cooperi*) as well (Nava et al., 2010b) and in southeast Brazil infestation of this rodent with both tick species is a common feature (Heijden et al., 2005; Perez et al., 2008).

All findings of *A. dubitatum* in Brazil were concentrated in the biogeographical provinces of Cerrado, Atlantic Forest, Parana Forest, and *Araucaria angustifolia* Forest (Nava et al., 2010b). At the same time, its distribution area is lesser than that of its principal host, the capybara, suggesting that environmental variables rather than hosts determine the distributional ranges of this tick species (Nava et al., 2010b).

Within its range, it is tick species related to areas prone to flooding (Szabó et al., 2007b; Queirogas et al., 2012). In fact, Queirogas et al. (2012) observed that *A. dubitatum* was privileged over *A. cajennense* by river margins exposed to flooding at least once a year. Thus, river margins in the urban areas without additional drier vegetation for *A. cajennense* maintained overwhelmingly *A. dubitatum* tick populations. At the same time, the role of *A. dubitatum* as vector of human diseases is undetermined but it is commonly associated to *R. bellii* (Pacheco et al., 2009), as far as known, a non-pathogenic *Rickettsia* species that does not belong to the SFG. Although not as aggressive as *A. cajennense, A. dubitatum* was already shown to bite humans (Labruna et al., 2007). Thus, the relationship of *A. cajennense, A. dubitatum*, and *R. rickettsii* deserves further investigation.

**Figure 2:**
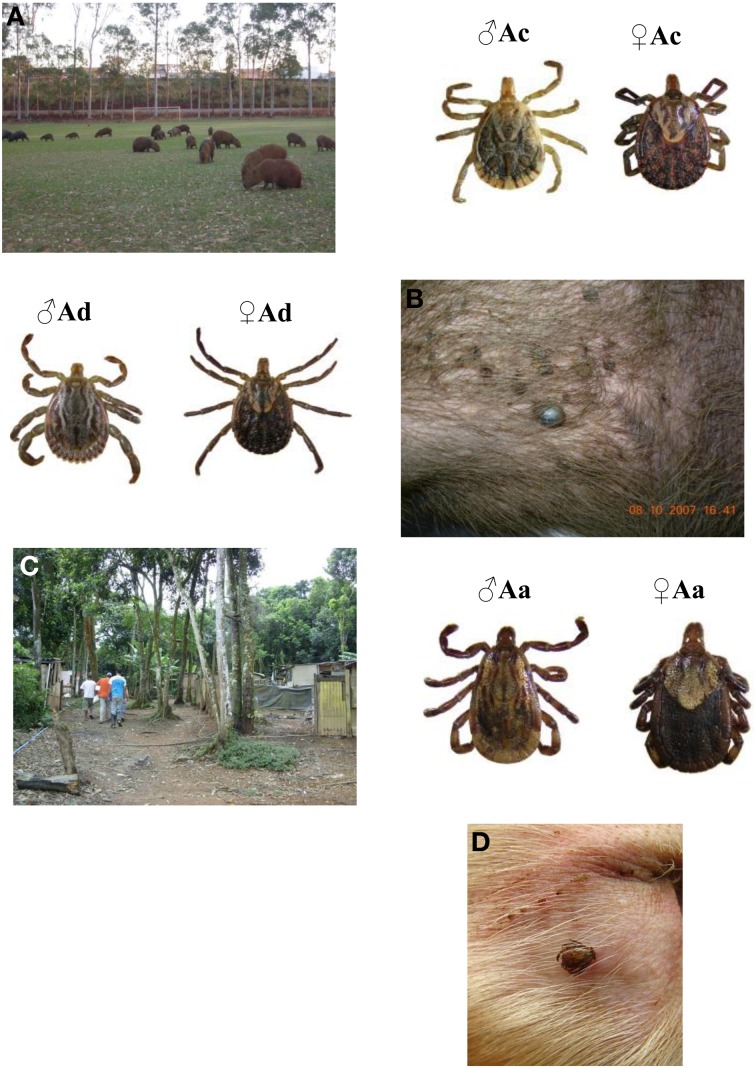
**Environmental backgrounds and ticks associated with Brazilian spotted-fever caused by human infection with *R. rickettsii* in Brazil**. Capybaras in anthropized area **(A)**. Tick-infested capybara **(B)**. Male and female adults of *Amblyomma cajennense* (Ac) and *Amblyomma dubitatum* (Ad). Endemic human settlement at the border of the Atlantic rainforest and metropolitan area of São Paulo **(C)**. Dog ear with an attached *Amblyomma aureolatum* **(D)**. Adult couple of *Amblyomma aureolatum* (Aa).

In summary, the ongoing major scenario for BSF is the occurrence of human cases associated to tick bites at anthropicized areas with capybaras (Figure 2). These areas close to water bodies, either riparian forest fragments or arborized small dams, are many times used for leisure activities and are landscape types in expansion in the country. In these areas both capybaras and their ticks flourish and human tick bites are a frequent event particularly by *A. cajennense*, a tick species very aggressive to humans. Such scenario is widespread in southeast Brazil encompassing many municipalities. In a few of these, BSF is endemic but still a rare event, probably because of the low infection rate of the vector tick *A. cajennense*. Since *R. rickettsii*-infected tick populations in nature depends on parasite feeding on a rickettsemic host such as a non-immune capybara, *A. cajennense* infection rate at a particular area seems to be a dynamic process; it decreases over time but can have focal uprising when such a host (amplifier host) is bitten by an infected tick. An unknown feature is the very initial source of *Rickettsia* in previously non-endemic areas and more detailed studies comparing ecological features of capybara–*A. cajennense* relationships in endemic and non-endemic area is mandatory.

Finally, experimental studies have shown that both opossums (*Didelphis aurita*) and domestic dogs may be competent amplifier hosts of *R. rickettsii* to ticks (*A. cajennnense* and *R. sanguineus*, respectively) (Horta et al., 2009; Piranda et al., 2011). Nevertheless, capacity of infection of solely 5% of *A. cajennense* places the opossum as secondary source of amplification to *R. rickettsii* among tick population. Furthermore, neither dogs nor opossums feed an important number of *A. cajennense* ticks when compared to capybaras. However, since both animal species are usually frequent in BSF-endemic areas, where they frequently become infested by immature stages of *A. cajennense*, their role in the ecology of the disease should be investigated deeper.

## Amblyomma aureolatum

A second but nonetheless important scenario associated to BSF in Brazil involves *A. aureolatum*. This scenario is much more constrained (Figure 1) because the environmental requirements and behavior of the vector tick have particular epidemiological features. Human BSF cases associated to *A. aureolatum* seem to occur when dogs are bitten by adult ticks during incursions into the rainforest and bring them back to human dwellings (Figure 2). From then on, two possible ways of human infection with *R. rickettsii* are supposed. In the main one, infected *A. aureolatum*, particularly males because they can remain on the dogs for several weeks, drop off from the dog accidentally (scratching, picked by humans) and bite humans (Pinter et al., 2004). The second possibility refers to infection of *R. sanguineus* ticks feeding on a dog parasitized by an infected *A. aureolatum* tick and will be discussed below. Although BSF cases caused in this scenario may have a wider distribution, most knowledge derives from studies at the metropolitan area of São Paulo (São Paulo State capital and other 38 municipalities) that is embedded in the Atlantic rainforest biome.

*A. aureolatum* is a Neotropical three-host tick, found in the eastern area of South America (Guglielmone et al., 2003). This tick species is associated with very humid habitat and cooler subtropical temperatures (Pinter et al., 2004). Thus, it is typically a tick from the Atlantic rainforest at higher altitudes in the Southeastern region (Sabatini et al., 2010) but can be found close to sea level in southern Brazil (Medeiros et al., 2011). Under laboratory conditions *A. aureolatum* is more susceptible to *R. rickettsii* infection than *A. cajennense* and is more efficient in maintaining the infection through 100% transstadial perpetuation, 100% transovarial transmission, as well as higher filial infection rates (Labruna et al., 2008, 2011c). At the same time, within BSF-endemic areas, infection rates of *A. aureolatum* by *R. rickettsii* have been reported to be around 1–10% (Pinter and Labruna, 2006; Ogrzewalska et al., 2012). Such relatively low infection rate might be explained, at least partially, by a deleterious effects caused by *R. rickettsii* in ticks (Niebylski et al., 1999; Labruna et al., 2011c).

In the case of *A. aureolatum*, no *R. rickettsii* amplifier host has been determined so far. In natural settings adults of *A. aureolatum* feed mainly on wild carnivore species (Guglielmone et al., 2003; Labruna et al., 2005b). The few host records for tick immature stages refer majorly to passerine birds, mainly the genus *Turdus* (Arzua et al., 2003; Ogrzewalska et al., 2012) and a few rodent species (Guglielmone et al., 2003). Additionally, a recent study (Ogrzewalska et al., 2012) observed that the BSF-endemic areas in São Paulo metropolitan area differed from the non-endemic areas by the presence of significantly smaller and more degraded forest patches in the former. Still the original amplifier source of the bacterium is undetermined and ecological features of endemic and non-endemic areas where *A. aureolatum* thrives should be compared further. For yet unknown reasons fatality rates of BSF at *A. aureolatum* transmission areas are higher (above 60%) and no obvious seasonal pattern for the disease can be detected (Angerami et al., 2012).

## Rhipicephalus sanguineus

Transmission of BSF by a third tick species, *R. sanguineus*, in Brazil is by now speculative but demands awareness for its potential. In fact this tick species is the main vector of *R. conorii agent* of the Boutonneuse fever in the Mediterranean basin and human *R. rickettsii* infection caused by *R. sanguineus* tick bites were already shown in the USA (Demma et al., 2005) and Mexico (Eremeeva et al., 2011). However, and again, care must be taken with tick populations involved with this rickettsiosis. It is by now well determined that South America has at least two genetically, morphologically, biologically, and geographically distinct species that have been treated under the taxon *R. sanguineus*: one is found in tropical and subtropical areas and the other in the south of South America (Southern Brazil, Uruguay, Chile, and Argentina) (Oliveira et al., 2005; Szabó et al., 2005; Moraes-Filho et al., 2011; Nava et al., 2012). Observations from here on refer solely to the tropical *R. sanguineus* populations of Brazil.

*R. sanguineus* is a tick, introduced in the country with colonization and is always associated to dogs (Szabó et al., 2005) and biting of other animals, including man, should be considered accidental. Up to our knowledge, this tick species has never been reported from natural or anthropized vegetation in Brazil. At the same time, it might attain high infestations levels at dog dwellings and thus close or at human dwellings (Labruna and Pereira, 2001; Guglielmone et al., 2006a), and it is found on dogs all over Brazil (Szabó et al., 2001, 2010; Dantas-Torres et al., 2004; Labruna et al., 2005a; Castro and Rafael, 2006; Soares et al., 2006). Although human *R. sanguineus* tick-biting in Brazil was reported before (Dantas-Torres et al., 2006), it is a rare event if one considers the frequent and close association of *R. sanguineus* and people. Importantly, it was shown in the laboratory that *R. sanguineus* is a competent *R. rickettsii* vector and, in opposition with that observed with *A. cajennense* and *A. aureolatum, R. rickettsii* did not elicit lethal effect on *R. sanguineus* (Piranda et al., 2011). Furthermore, naturally occurring infection of *R. sanguineus* with *R. rickettsii* in Brazil was already observed in BSF endemic areas by molecular tools and isolation in cell culture (Cunha et al., 2009; Gehrke et al., 2009; Moraes-Filho et al., 2009; Pacheco et al., 2011; Ogrzewalska et al., 2012).

**Figure 3:**
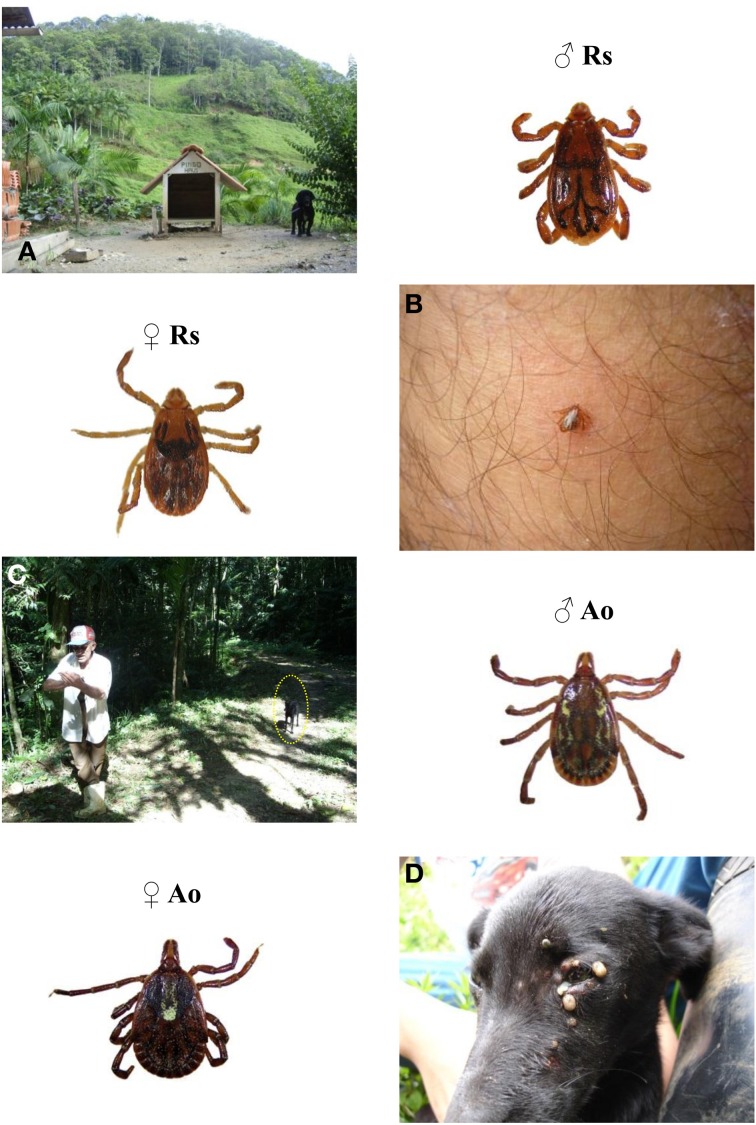
**Environments with potential for dog and human infestation with ticks associated to spotted-fever in Brazil**. Dog restrained to its house providing conditions for the maintenance of *Rhipicephalus sanguineus* infestation but with potential for infestation with *Amblyomma ovale* or *Amblyomma aureolatum* in the nearby forest **(A)**. *Rhipicephalus sanguineus* bite of a human leg **(B)**. Adult couple of *Rhipicephalus sanguineus* (Rs). Atlantic rainforest with high *Amblyomma ovale* prevalence **(C)**. Dog infestation with *Amblyomma ovale* ticks at the same area **(D)**. Adult couple of *Amblyomma ovale* (Ao).

Although human BSF infection transmitted by *R. sanguineus* in Brazil is still unproven, there is a very likely scenario for that. *R. sanguineus* ticks are mostly urban in the country (Szabó et al., 2001; Labruna, 2004; Labruna et al., 2005a) and thus held apart from *R. rickettsii* sources. However, there are many free-ranging dogs either ownerless or kept unrestrained by owners. In many instances, these animals wander between urban and natural areas and are infested with tick species from both environments (Figure 3) (Moraes-Filho et al., 2009; Queirogas et al., 2010). Under such conditions, *R sanguineus* ticks may feed on dogs previously or concomitantly harboring *R. rickettsii*-infected *A. cajennense* or *A. aureolatum* ticks and be infected during rickettsemia. In fact, dog infestation with *R. rickettsii*-infected *R. sanguineus* alongside infected *A. aureolatum* ticks in endemic areas has been recently reported (Moraes-Filho et al., 2009; Ogrzewalska et al., 2012). This very likely bridge of *R. rickettsii* to *R. sanguineus* infestation sites opens the gate for dissemination of the bacterium in ticks that apparently do not suffer its lethal effect and may propagate unrestrained. In this regard it is interesting to observe that in endemic areas *R. rickettsii* infection prevalence tend to be higher in *R. sanguineus* than *A. aureolatum* (Moraes-Filho et al., 2009; Ogrzewalska et al., 2012). Thus, we can suppose that at sites with high *R. sanguineus* (and dog) densities, *R. rickettsii* infection introduced by *Amblyomma* species may be overshadowed by infection of *R. sanguineus* populations. Fortunately, *R. sanguineus* is not as aggressive to humans in Brazil, and transmission might occur only in the case of occasional tick bites or by crushing of ticks picked from animals, more likely to people who handle dogs frequently.

*Amblyomma* and *Rhipicephalus* mixing may be particularly relevant at places with a constant influx of wandering dogs of unknown origin such as Zoonosis Control Centers and dog shelters. In a Zoonosis Control Center in a BSF-endemic area of Minas Gerais State, high prevalence of *R. rickettsii*-infected *R. sanguineus* was observed (Pacheco et al., 2011). Furthermore, recent death of four employees working at a dog shelter was attributed to BSF in Rio de Janeiro. In this case, the only tick species found at that location was *R. sanguineus*, and 97% of 117 tested dogs were seropositive to *R. rickettsii* antigen (Costa et al., 2012). Thus, such scenarios for *R. sanguineus* transmission of BSF should be deeply investigated.

## *Amblyomma ovale* and atlantic rainforest *Rickettsia*

Only at the end of the last decade did the diagnoses of a second human tick-borne spotted-fever rickettsiosis occurred in the country (Spolidorio et al., 2010). The agent was a novel SFG strain closely related to *R. africae, R. parkeri*, and *R. sibirica* and caused a febrile illness but milder than BSF. The causative *Rickettsia* strain was named Atlantic rainforest due to the environment it was found (Sabatini et al., 2010; Spolidorio et al., 2010), but species definition was at the time controversial. A second clinical case due to this novel agent was subsequently reported in another region of the country (Silva et al., 2011), highlighting the possibility that Atlantic rainforest rickettsiosis could be much more frequent than currently known.

Atlantic rainforest *Rickettsia* strain was shown to be strongly associated with *A. ovale* ticks from the Atlantic rainforest and seems to have a wide range, at least in the south–southeastern Atlantic coast of Brazil (Figure 1) (Sabatini et al., 2010; Medeiros et al., 2011; Szabó et al., 2013). Within the studied areas, *A. ovale* tick populations attain infection levels of around 10% (Sabatini et al., 2010; Szabó et al., 2013). Importantly, adult of this tick species attaches to and feeds readily on dogs and is thus frequently reported from dogs kept unrestrained in rural areas close to natural environments (Szabó et al., 2001, 2010, 2013; Labruna et al., 2005a; Sabatini et al., 2010). Moreover, *A. ovale* adult tick human bite is frequent (Labruna et al., 2005a; Guglielmone et al., 2006b; Szabó et al., 2006).

Under natural conditions, *A. ovale* is a three-host tick species with adults parasitizing carnivores whereas rodents are main hosts for immature feeding stages (Guglielmone et al., 2003; Labruna et al., 2005b; Szabó et al., 2013). *A. ovale* has wide distribution (Neotropical–Neartic) (Guglielmone et al., 2003) and has a surprising ecological plasticity being found in several Brazilian biomes, including Pantanal (Pereira et al., 2000), Amazon (Labruna et al., 2005a), Atlantic rainforest (Szabó et al., 2009), and Cerrado (Szabó et al., 2007a). Comparison of these populations from diverse environments is warranted to better define their relationships as well as vectoring capabilities.

Up to now, SFG *Rickettsia* infection of *A. ovale* was associated only with Atlantic rainforest populations where adult ticks were shown to quest on vegetation in high numbers (Szabó et al., 2013). *Rickettsia* amplifier host or reservoir in the forest has not been determined so far, but a small rodent, *Euryoryzomys russatus*, was shown to be an important host for *A. ovale* immatures as well as attained high seroconversion prevalence and high specific titers in one study (Szabó et al., 2013). Thus, humans can be *Rickettsia* infected if bitten by ticks during incursions into the forest or by ticks detached from the dogs. In the latter case, human infection is supposed to occur faster as *Rickettsia* reactivation (Hayes and Burgdorfer, 1982) should have occurred during feeding on the first host.

Importance of dogs in the epidemiology of the Atlantic rainforest rickettsiosis goes beyond possible *Rickettsia* reactivation in *A. ovale* ticks. Such hosts, that frequent the forest in endemic areas, are chiefly infested with *A. ovale* ticks (Figure 3), many *Rickettsia* infected, and all these dogs seroconvert, attaining very high titers against *R. parkeri* antigens (Sabatini et al., 2010; Medeiros et al., 2011; Szabó et al., 2013). Furthermore, Atlantic rainforest *Rickettsia* was detected by molecular tools in *A. aureolatum* and *R. sanguineus* on dogs co-infested with *A. ovale* ticks (Sabatini et al., 2010; Medeiros et al., 2011; Szabó et al., 2013). Thus, a role for *A. aureolatum* and *R. sanguineus* on dogs in the epidemiology of the disease also deserves investigation.

By this time, it can be supposed that many human cases formerly considered as mild BSF (Angerami et al., 2009) or other febrile illness were in fact Atlantic rainforest rickettsiosis. Undoubtedly a careful analysis of tick-borne human febrile illness along the Brazilian coast is mandatory to evaluate the range of the disease.

## Concluding remarks

As depicted from information above, agents of human tick-borne rickettsiosis in Brazil originate from wildlife. Unfortunately, the very initial source of such pathogenic *Rickettsia* is not determined and thus epidemiology of human infections has knowledge gaps. Whatever the origin of pathogenic *Rickettsia*, human activities can be blamed for amplification of both wildlife host (capybara) and tick infections as well as bridging from natural environment to human dwellings (unrestrained dogs). In all such cases, likelihood of human infection increases several fold. Nonetheless, recognition of the ecological background of each rickettsiosis is a major step to provide diagnosis, treatment and preventive measures. In this regard, increased capybara and *A. cajennense* populations at locations with human activities and dogs with access to wildlife environment are key features in the infection of human beings. Unfortunately, capybara populations are increasing in urban and peri-urban areas and control possibilities face a complex situation involving technical, ethical, and political aspects, as well as society issues. On the other hand, limiting dog access to wildlife areas seems to be a more feasible measure but which should rely on educational ground of animal owners as well as control of free-roaming ownerless dog populations.

We herein put together available information on tick ecology and *Rickettsia* to build the most probable epidemiology of known Rickettsiosis in Brazil. However, existing knowledge is still overwhelmingly restricted to southeast Brazil, and even in this region, it is not enough to provide a final picture. In this context, proper determination of tick species and their distribution in every geographic region is a prerequisite to unfold epidemiology of Rickettsiosis. Furthermore, there is an ongoing discovery of other *Rickettsia* species in the country and novel human Rickettsiosis with particular epidemiology might be revealed. A complicating factor is the fast man-induced landscape changes that alter existing host–tick relationships creating new scenarios for tick-borne diseases. Nonetheless, information gathered here is a good starting point to evaluate Rickettsial disease epidemiology in other geographical regions of the country as well.

### Conflict of interest statement

The authors declare that the research was conducted in the absence of any commercial or financial relationships that could be construed as a potential conflict of interest.
